# Cyanoacrylate Tissue Adhesives in Peri‐Implant Surgery: A Case Report and Surgical Technique

**DOI:** 10.1002/ccr3.71553

**Published:** 2025-12-02

**Authors:** Patricio Vicencio, Dusan Marinkovic, Hans Guler, Claudio Miranda

**Affiliations:** ^1^ Escuela de Odontología, Facultad de Medicina Clínica Alemana Universidad Del Desarrollo Santiago Chile

**Keywords:** cyanoacrylates, dental implants, periodontal surgery, tissue adhesives

## Abstract

Cyanoacrylate tissue adhesives have been proposed as an adjunct or alternative to sutures in periodontal and peri‐implant surgery, offering hemostasis, bacterial sealing, and improved wound healing. However, their clinical application remains scarcely documented. This case describes the adjunctive use of n‐butyl‐2‐cyanoacrylate in combination with sutures for donor site stabilization during peri‐implant soft tissue grafting in a 67‐year‐old female patient rehabilitated with implants in the maxillary right quadrant. A free gingival graft was harvested from the palatal premolar region and secured with sutures and cyanoacrylate adhesive, while the recipient site received a split‐thickness flap under local anesthesia. Healing was uneventful, with complete graft integration and stable keratinized mucosa formation in the donor site after 30 days. No inflammation, infection, or necrosis was observed, and postoperative discomfort was minimal. The adjunctive use of cyanoacrylate provided rapid hemostasis, effective wound protection, and enhanced patient comfort, suggesting a simple and clinically feasible approach to soft tissue management around implants. However, these findings are based on a single case and should be interpreted descriptively.

## Key Clinical Message

The adjunctive use of cyanoacrylate with sutures may provide a practical means to enhance hemostasis and comfort at the palatal donor site, supporting stable clot formation and uneventful healing after free gingival graft harvesting.

## Graphical abstract

Clinical view of wound closure using cyanoacrylate tissue adhesive.

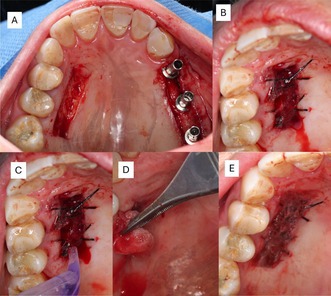

## 1 Introduction

Periodontal and peri‐implant surgeries require precise soft tissue management to achieve stable healing and minimize complications. Conventional sutures remain the standard for wound closure; however, they may prolong surgical time and are associated with plaque accumulation and local inflammation [1, 2]. These limitations have encouraged the exploration of adjunctive methods to improve wound stabilization and postoperative comfort.

Cyanoacrylate‐based tissue adhesives have been introduced as an adjunct or alternative to sutures due to their hemostatic, sealing, and bacteriostatic properties [3, 4]. Upon contact with moisture, cyanoacrylate rapidly polymerizes, forming a thin, protective layer that stabilizes the clot and isolates the wound from mechanical and bacterial irritation. Its use may therefore enhance soft tissue healing while reducing the need for extensive manipulation.

The aim of this case report, prepared in accordance with the CARE (Case Report Guidelines), was to present a surgical technique that integrates n‐butyl‐2‐cyanoacrylate as an adjunct to conventional sutures during peri‐implant soft tissue augmentation. This approach aimed to control persistent bleeding at the palatal donor site, protect the surgical area, enhance wound stability and early healing, and improve patient comfort.

## 2 Patient Information

This case report was conducted in accordance with international ethical standards, including the Declaration of Helsinki. Ethical approval was obtained from the Scientific Ethical Committee of the Universidad del Desarrollo (Approval Code: 2025‐64), where the clinical procedure was performed, evaluated, and approved for the case of a 67‐year‐old female patient with no relevant medical history or systemic comorbidities. She denies any risk habits such as smoking, alcohol consumption, or drug use, and has a dental history of tooth loss secondary to caries. Five months earlier, she underwent surgery for the placement of dental implants in sites 12, 13, and 14, with no intraoperative or postoperative complications.

## 3 Clinical Findings

**FIGURE 1:**
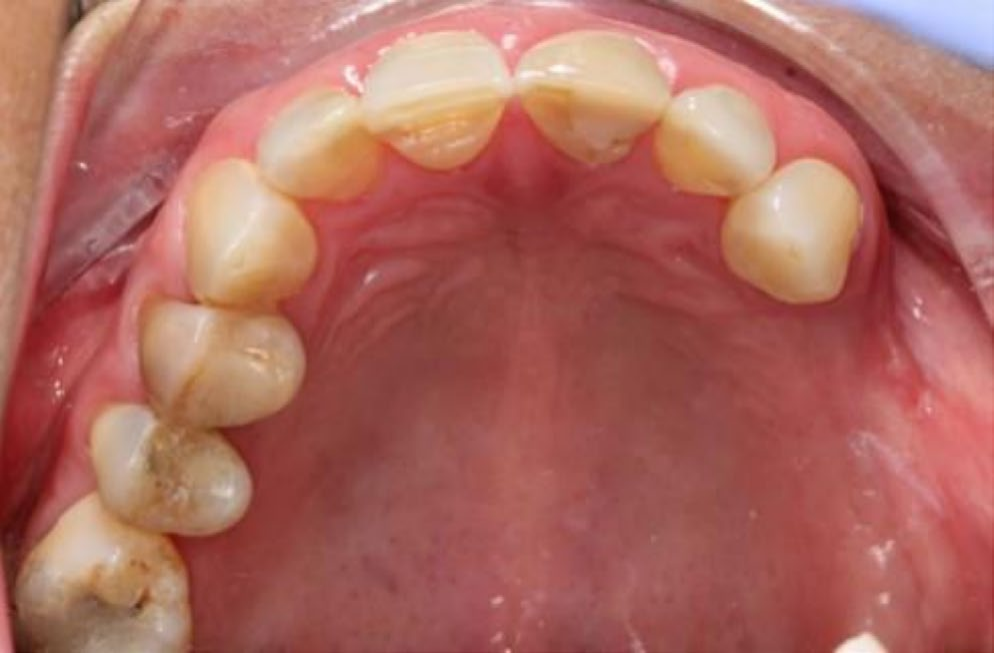
Initial clinical situation. The alveolar mucosa near the ridge area and over the submerged implants (unconnected) in regions 12, 13, and 14 appears thin, mobile, and nonkeratinized, with a reddish color and absence of a defined mucogingival junction. The lack of vestibular keratinized gingiva and the presence of a shallow vestibule suggest limited soft tissue stability, which may compromise plaque control and peri‐implant health.

The general diagnosis was partial edentulism in the upper right quadrant, with missing teeth in positions 12, 13, and 14, where submerged implants had been previously placed. The periodontal diagnosis corresponded to periodontal health on a reduced periodontium in the remaining dentition, associated with a mucogingival defect characterized by the absence of keratinized mucosa around the implant sites (Figure 1). This condition represents a less favorable prognosis for implant‐supported rehabilitation due to the higher susceptibility to peri‐implant inflammation and the increased difficulty in maintaining adequate plaque control. Additionally, the patient reported pain and discomfort during toothbrushing, even when using a soft‐bristled toothbrush.

## 4 Therapeutic Intervention

A free gingival graft from the palate was performed to increase the peri‐implant keratinized mucosa in implants 12, 13, and 14. The donor site was closed using simple hemostatic sutures complemented by cyanoacrylate tissue adhesive for additional stabilization and hemostatic control, with the aim of improving oral hygiene, soft tissue stability, and postoperative comfort. The chronological sequence of the clinical events is summarized in Figure 1, which illustrates the main stages from baseline assessment to the 30‐day postoperative follow‐up. The timeline shows the surgical intervention, early healing phase, suture removal, and final evaluation, highlighting the progressive and uneventful healing process observed at both donor and recipient sites.

## 5 Surgical Procedure

Local anesthesia was achieved with 2% lidocaine containing epinephrine 1:100,000 (two 1.8 mL cartridges), administered at both donor and recipient sites. After antisepsis and isolation, the recipient site was prepared for soft‐tissue augmentation. A split‐thickness flap was elevated to expose the vascular connective bed, with the superficial epithelium and loose connective tissue gently removed to ensure adequate perfusion and intimate graft adaptation.

The free gingival graft was harvested from the palatal region corresponding to the premolars, following anatomical safety guidelines to minimize the risk of injury to the greater palatine artery. The graft measured approximately 10 × 8 × 1.5 mm (length × width × thickness) and was obtained at split thickness using a scalpel blade No. 15C. The graft was immediately placed in sterile saline to maintain hydration until transfer to the recipient site.

The donor site was managed using a hybrid closure protocol combining sutures and tissue adhesive. Four simple interrupted cross‐pattern hemostatic sutures (3‐0 silk) were placed at 2.5–3.0 mm intervals to approximate the wound margins without tension and achieve mechanical stability of the clot. Before applying the adhesive, the wound was thoroughly irrigated with approximately 5 mL of sterile saline using a syringe, with the objective of removing excess blood and creating a uniformly moist surface, which promotes controlled polymerization of cyanoacrylate through the hydroxyl ion–mediated anionic reaction.

Subsequently, n‐butyl‐2‐cyanoacrylate (Periacryl 90, GluStitch Inc., Delta, BC, Canada) was carefully applied along the suture line using a fine applicator tip to dispense approximately 0.05 mL of adhesive in a single continuous layer. After Periacryl application, the area was gently moistened with a 10 × 10 mm sterile gauze pad soaked in saline solution, promoting uniform polymerization of the adhesive. Polymerization of the first layer began within 10–20 s and achieved complete surface hardening between 50 and 70 s.

The process of applying Periacryl was then repeated until the area was completely covered, again followed by saline application; in total, the Periacryl and saline application sequence was performed three times. The final inspection confirmed a stable and hemostatic closure, with no excess adhesive or tension on the soft tissues. The total operative time required for donor site closure was approximately 10–6 min for suture placement and 4 min to fully cover the wound with a hardened Periacryl layer. Complete hemostasis of the surgical wound was achieved at 10 min, measured from the beginning of the suturing phase to the application of the final layer of biomaterial. The total volume of adhesive used was 0.2 mL, corresponding to the entire content of the single‐dose vial.

At the recipient site, the graft was positioned and secured using 5‐0 nylon monofilament sutures (three simple interrupted stitches: one central and two lateral), ensuring intimate adaptation and immobility. The remaining mucosal flap was apically repositioned without tension and stabilized with light approximation sutures as needed to facilitate optimal integration and maintain vestibular depth.

**FIGURE 2:**
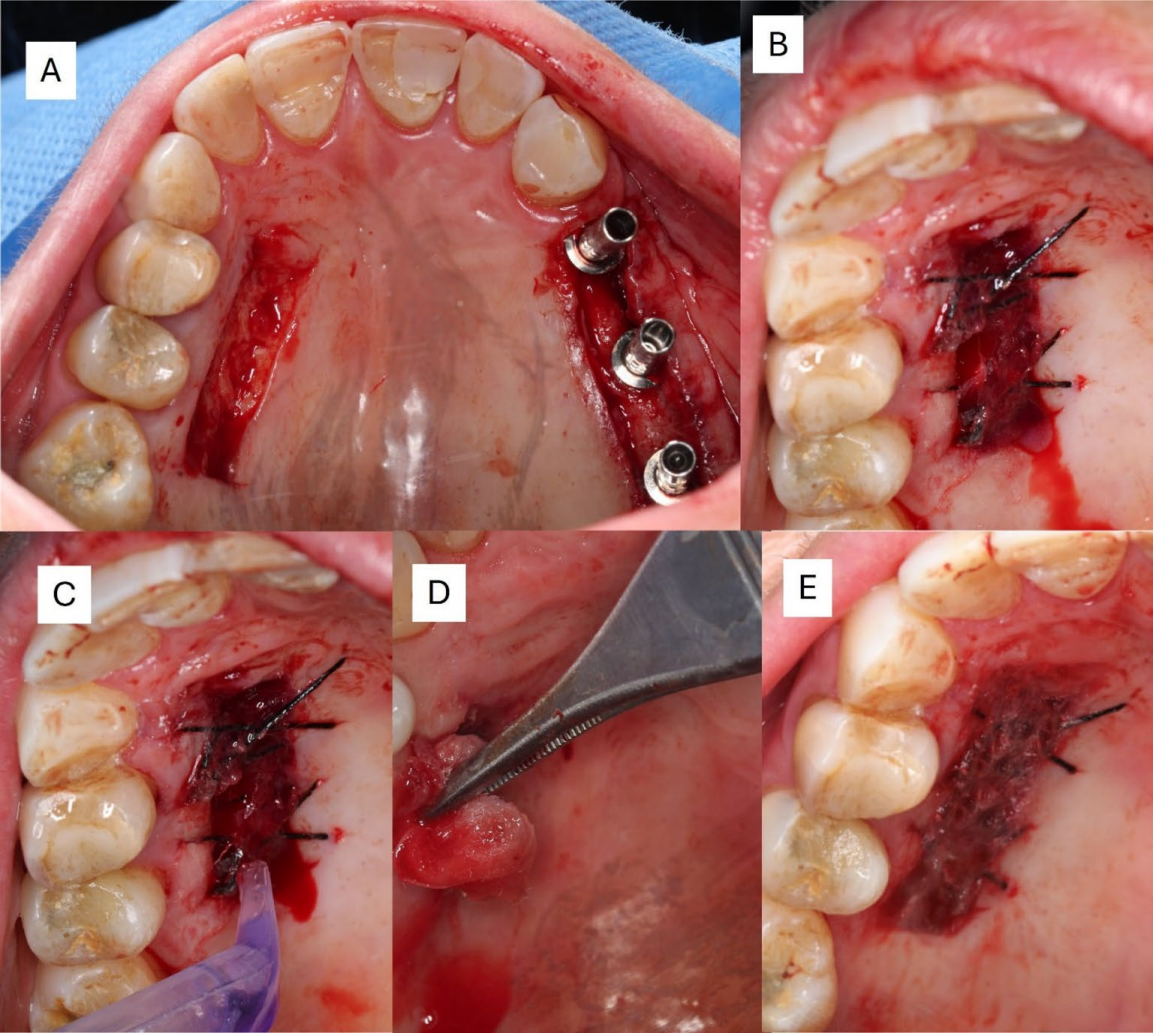
Intraoperative stages of cyanoacrylate tissue adhesive application (A–E). (A) The donor site is shown after removal of the free gingival graft, revealing a healthy bleeding bed with uniform coloration and well‐defined margins. The recipient site displays healing abutments previously installed. (B) The donor site with sutures in place shows active bleeding and a bright red tissue surface, indicating adequate vascularization and viability of the flap. (C) Application of cyanoacrylate tissue adhesive in single‐dose format over the sutured area. The adhesive begins to form a thin layer that stabilizes the clot. (D) Sterile saline solution is applied over the cyanoacrylate layer to accelerate polymerization. The tissue exhibits gradual hemostasis and reduction in bleeding intensity. (E) Complete polymerization of the cyanoacrylate tissue adhesive is observed, forming a uniform, glossy surface barrier with cessation of bleeding. The underlying tissue shows good color and no signs of ischemia or necrosis, suggesting adequate perfusion and protection of the wound.

The surgical workflow including anatomical orientation, graft dimensions, suture configuration, and adhesive application sequence, is shown in Figure 2A–E, which illustrate the procedural timeline from graft harvesting to final palatal closure.

## 6 Postoperative Care

Painkillers (ibuprofen 600 mg every 8 h and acetaminophen 1 g every 8 h for 3 days), antibiotics (amoxicillin 500 mg every 8 h for 7 days), and rinsing with 0.12% chlorhexidine twice a day for 2 weeks were prescribed. The patient was instructed to avoid brushing the treated area for 10 days. A 30‐day follow‐up is sufficient to assess healing of the palatal donor site, as epithelialization, initial tissue maturation, and clot stability are typically achieved within this period [2, 5, 6].

## 7 Follow‐Up and Outcomes

**FIGURE 3:**
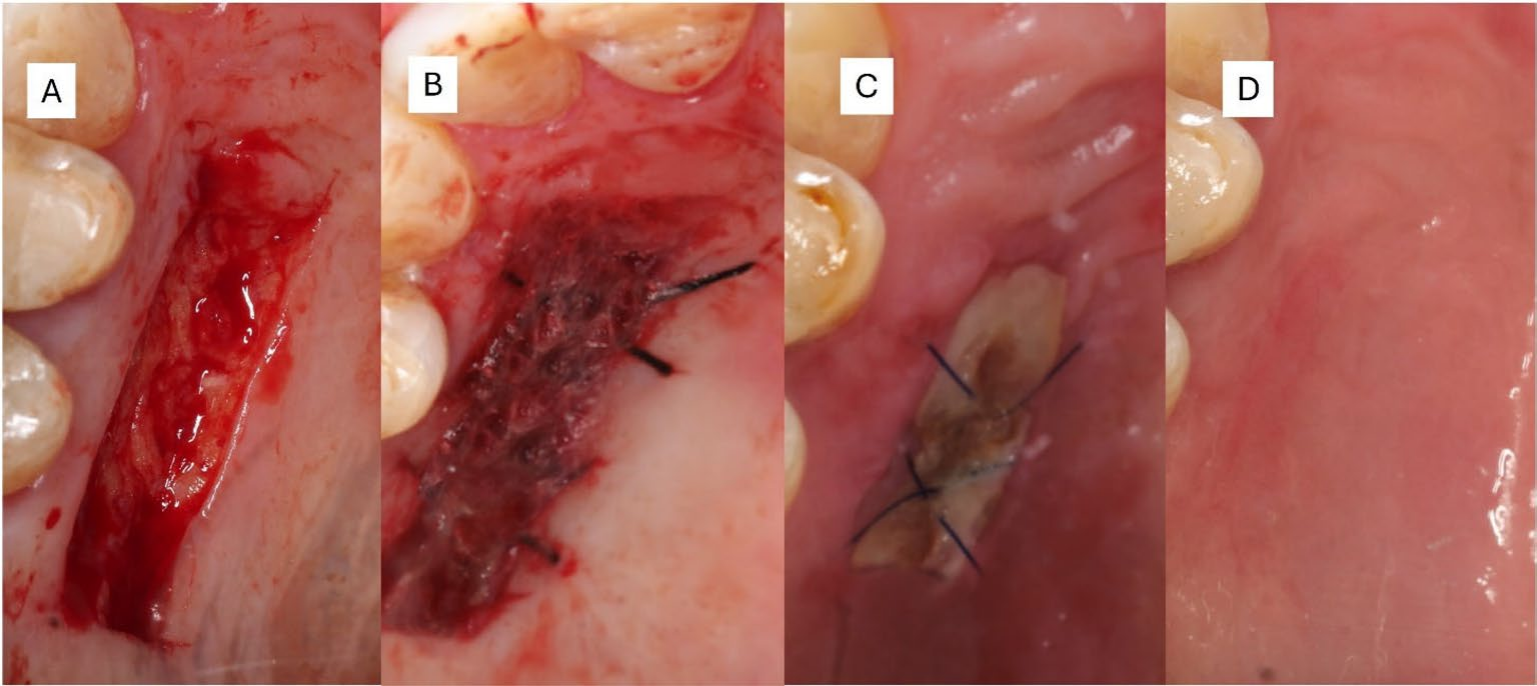
Healing process at the donor site (A–D). (A) Initial wound immediately after graft harvesting, with a bright red color consistent with a vital, well‐vascularized connective tissue bed. (B) Intraoperative view following cyanoacrylate application shows a shiny, translucent film covering the wound, indicating complete polymerization. Mild erythema at the edges reflects normal early inflammatory response. (C) At 14 days, the wound displays partial epithelialization with decreased erythema and a residual hardened cyanoacrylate layer protecting the underlying tissue. The color shifts from red to pink, denoting granulation and revascularization. (D) At 30 days, complete healing is evident with restoration of mucosal continuity, uniform pink coloration, and absence of inflammation or scar formation, consistent with healthy epithelial maturation and tissue integration.

**FIGURE 4:**
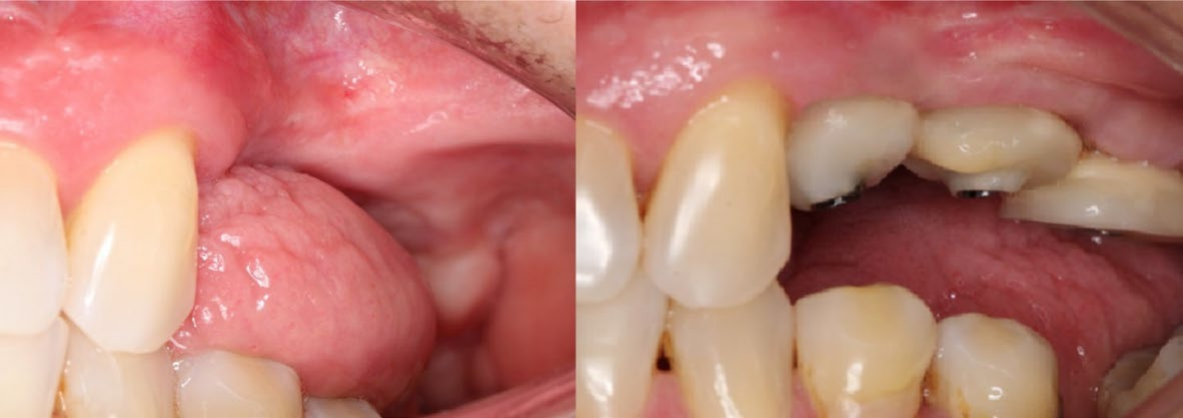
Pre‐ and postoperative peri‐implant soft tissue condition. The left image shows the preoperative situation, characterized by limited keratinized tissue and a high frenulum insertion close to the alveolar ridge. The mucosa appears red, thin, and movable, reflecting poor keratinization and susceptibility to trauma. The right image, at 30 days, shows temporary restorations shaping the emergence profile. A thick, well‐defined band of peri‐implant keratinized mucosa with light pink color and firm texture is evident, indicating successful graft integration, maturation, and functional stability of the peri‐implant soft tissues.

The chronological sequence of healing is illustrated in Figure 3A–D, depicting the intraoperative phase, immediate postoperative condition, and evaluations at 14 and 30 days. At 7 days, the donor sites exhibited residual adhesive integrity and tissue stability supported by tension‐free sutures, with no clinical evidence of bleeding, dehiscence, or delayed healing. Sutures were removed at 14 days (Figure 3C). By 30 days, complete epithelialization of the donor area was achieved, with intact mucosal continuity and no signs of inflammation or infection (Figures 3D and 4).

**FIGURE 5:**
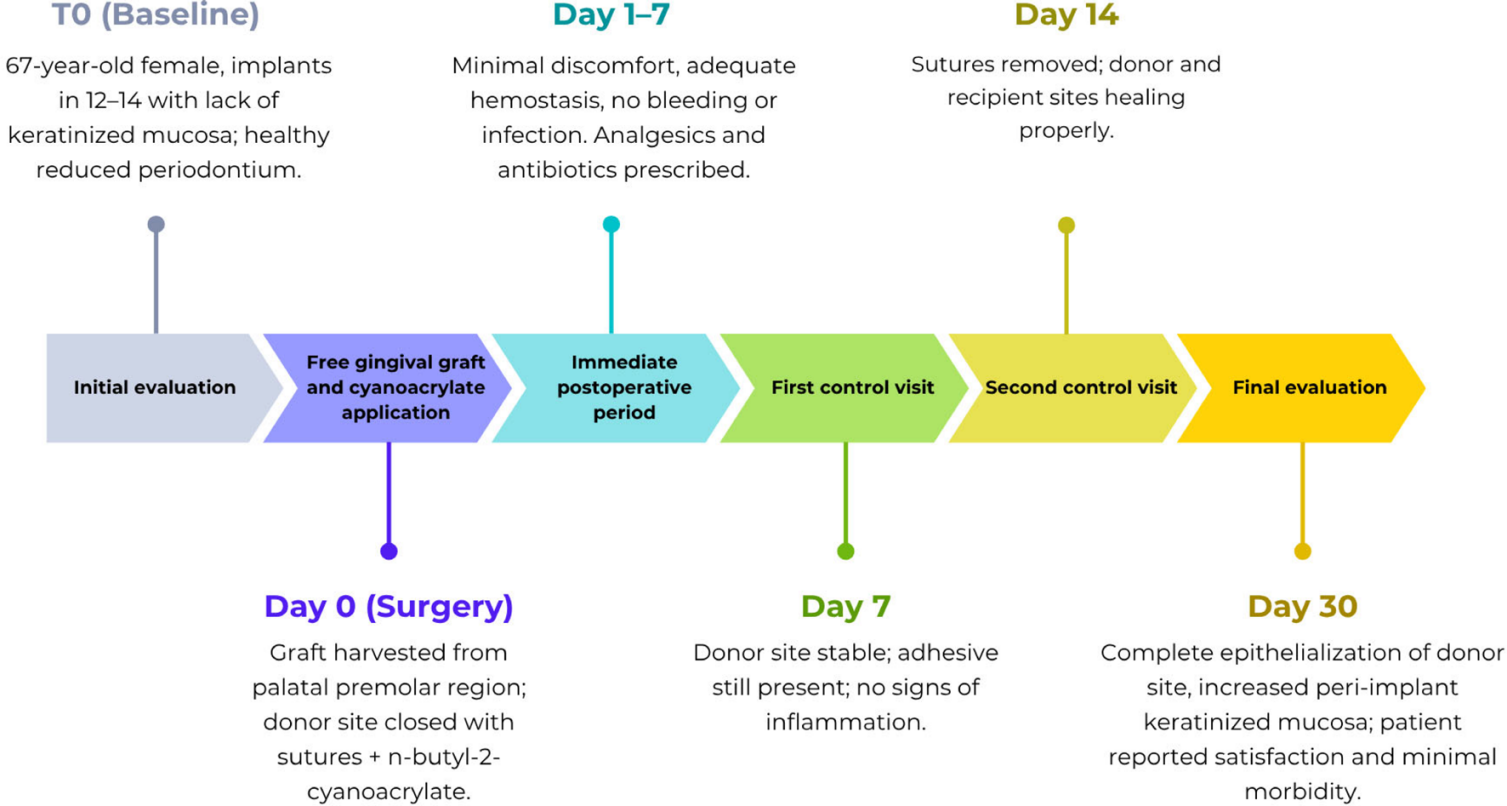
Clinical timeline summarizing the main stages of the case, from baseline assessment to 30‐day follow‐up.

Quantitative assessment of the recipient sites revealed an increase in both the width and thickness of keratinized mucosa, from baseline mean values of 2.3 and 1.8 mm to 4.8 and 2.9 mm at 30 days, respectively, as determined by transgingival probing using a standardized periodontal probe. At days 14 and 30, plaque accumulation was minimal, and bleeding on probing was observed in only a limited proportion of sites (12%–14%), reflecting effective plaque control and a minimal inflammatory response. The clinical timeline is summarized in Figure 5.

At the donor sites, complete epithelialization was clinically confirmed at 30 days, following a transitional phase of fibrin membrane coverage at 7 days. No cases of spontaneous bleeding, wound dehiscence, or infection were recorded throughout the observation period.

Patient‐reported outcomes revealed mild postoperative discomfort at the donor site, with a mean VAS pain score of 4/10 during the first 48 h and 1/10 by day 7. Pain intensity was self‐assessed by the patient using the Visual Analogue Scale (VAS), at 24 and 48 h, and on postoperative days 3 and 7, to ensure standardized and reproducible evaluation of subjective discomfort. Analgesic medication was required only during the first 48 h postoperatively, administered as an adjunct to routine postoperative care, given the minimal discomfort reported by the patient. The antibiotic regimen was completed as prescribed, with no adverse reactions or intolerance observed.

## 8 Discussion

Cyanoacrylates are biocompatible tissue adhesives with well‐documented hemostatic and bacteriostatic properties, as well as the ability to promote wound healing, which has driven their use in oral surgery since their FDA approval in 1998 [1, 2, 7]. Their rapid polymerization in the presence of moisture allows the formation of a solid matrix that provides a firm yet flexible mechanical bond between tissue margins, even in the challenging humid environment of the oral cavity 6–9. Among the most widely used formulations are n‐butyl‐2‐cyanoacrylate and 2‐octyl‐cyanoacrylate, which differ in flexibility, tensile strength, and degradation rate [8, 9]. From a biological perspective, cyanoacrylates serve as protective barriers that prevent bacterial contamination, ensure rapid hemostasis, and support controlled, atraumatic healing. The polymerization of n‐butyl‐2‐cyanoacrylate triggers an exothermic reaction, generating a thin polymer film that seals the wound and stabilizes the clot. Its biocompatibility and gradual resorption permit normal healing dynamics, such as epithelial migration and connective tissue remodeling, to proceed without interference. The spontaneous detachment within 7–10 days eliminates the need for manual removal, and both clinical and histological studies report a minimal inflammatory response when correctly applied [2, 5, 6, 10], although occasional localized reactions have been observed at low frequency (0.5%–14%) [5, 10].

When used in conjunction with conventional sutures, cyanoacrylates allow for enhanced hemostasis, improved margin sealing, and potential reduction in dehiscence, inflammation, and postoperative pain, in addition to shortening surgical time [1, 2, 6, 9, 10]. However, in areas of high tension or mobile mucosa, sutures remain superior due to their ability to withstand greater tensile forces [1, 10]. Limitations of cyanoacrylate include the need for a relatively dry environment, avoidance of excessive tension, risk of premature polymerization, associated costs, and a learning curve for correct application [5, 11].

In periodontal and peri‐implant procedures, cyanoacrylate has often been studied as an alternative to conventional suturing, with multiple randomized controlled trials (RCTs) evaluating its efficacy. Most of these studies have focused on its use as a stand‐alone closure method, either to maintain graft stability or reduce postoperative pain at donor sites, or for flap closure in oral surgeries such as third molar extraction, where flap tension is a critical factor. In the context of free peri‐implant gingival grafts, cyanoacrylates offer significant clinical advantages by reducing tissue manipulation, improving clot stability, and promoting graft integration, which is particularly useful in patients with limited keratinized tissue [2, 9, 12].

While most previous studies have evaluated cyanoacrylate as an alternative to sutures, the present report focuses on its adjunctive use, combining both techniques to optimize wound stability and patient comfort [5, 10]. The present case introduces cyanoacrylate as an adjunct to simple hemostatic sutures at the palatal donor site. This dual‐closure approach addresses specific challenges of this anatomical area, where hemostasis may remain incomplete after suturing alone, and the initial clot is particularly vulnerable to disruption. The application of cyanoacrylate over sutures, coupled with accelerated polymerization using sterile saline, provided immediate hemostasis and formed a solid protective matrix over the wound margins. This hybrid closure may have contributed to less tissue manipulation, reduced postoperative discomfort, and the absence of additional manual removal. At 30 days postoperatively, the palatal donor site demonstrated complete, uneventful healing, with a stable increase in peri‐implant keratinized mucosa. These outcomes are highly favorable and comparable to those reported in RCTs employing single closure techniques, both in terms of soft‐tissue healing and absence of infection.

In the present case, the staged approach combining mechanical stabilization through sutures with chemical sealing achieved by cyanoacrylate polymerization, appeared to provide effective control of bleeding and adequate protection of the surgical wound. The use of a controlled polymerization protocol may have helped prevent local heat generation and contributed to improved postoperative comfort. Moreover, the resulting adhesive film functioned as a protective barrier that likely reduced external contamination and mechanical trauma during the early stages of wound healing.

From a technical and clinical perspective, this case provides a reproducible protocol for palatal donor‐site management that emphasizes rapid hemostasis and clot protection. It is particularly relevant for patients presenting a reduced periodontal support around teeth or a limited zone of keratinized mucosa around implants, conditions associated with a less favorable prognosis for implant‐supported rehabilitation. By minimizing tissue manipulation, shortening surgical time, and reducing postoperative discomfort, this dual‐closure approach may improve patient acceptance and facilitate oral hygiene in high‐risk cases. Overall, the combination of sutures and cyanoacrylate adhesive represents a clinically relevant refinement of conventional closure methods, demonstrating effective bleeding control and predictable healing at the palatal donor site.

The findings are descriptive rather than analytical, reflecting clinical observations not supported by statistical or comparative evaluation. The unavailability of histologic correlation limits the interpretability of the results and should be considered when assessing the reported tissue behavior.

## 9 Conclusion

Within the limitations of a single case, the use of cyanoacrylate tissue adhesive as an adjunct to conventional sutures may contribute to improved wound stability and patient comfort at palatal donor sites during peri‐implant soft tissue augmentation. This approach could represent a practical alternative for reducing postoperative discomfort and facilitating early healing. However, further clinical studies are necessary to substantiate these observations. We highlight the invitation to conduct specific randomized controlled trials (RCTs) to evaluate the efficacy and safety of this intervention, with the aim of generating robust evidence to support its clinical application.

## Author Contributions

**Patricio Vicencio:** conceptualization, data curation, formal analysis, investigation, methodology, project administration, resources, software, validation, visualization, writing – original draft, writing – review and editing. **Dusan Marinkovic:** conceptualization, data curation, formal analysis, funding acquisition, investigation, methodology, project administration, resources, software, supervision, validation, visualization, writing – original draft, writing – review and editing. **Hans Guler:** conceptualization, data curation, formal analysis, funding acquisition, investigation, methodology, project administration, resources, software, validation, visualization, writing – original draft, writing – review and editing. **Claudio Miranda:** conceptualization, data curation, formal analysis, funding acquisition, investigation, methodology, project administration, resources, software, validation, visualization, writing – original draft, writing – review and editing.

## Funding

The authors have nothing to report.

## Disclosure

Permission to reproduce material from other sources: No material from other sources was reproduced in this article.

## Ethics Statement

This study was conducted in accordance with the ethical standards of the institutional and/or national research committee and with the 1964 Helsinki declaration and its later amendments. Ethical approval was obtained from the Comité Ético‐Científico de la Universidad del Desarrollo (Approval Code: 2025–64).

## Consent

Written informed consent was obtained from all patients (or their legal guardians) for inclusion in the study and for publication of clinical data and images.

## Conflicts of Interest

The authors declare no conflicts of interest.

## Data Availability

The data that support the findings of this study are available on request from the corresponding author. The data are not publicly available due to privacy or ethical restrictions.
